# Genetic Diversity and Population Structure of *Anopheles funestus* in Western Kenya Based on Mitochondrial DNA Marker *COII*

**DOI:** 10.3390/insects14030273

**Published:** 2023-03-09

**Authors:** Isaiah Debrah, Kevin O. Ochwedo, Wilfred O. Otambo, Maxwell G. Machani, Edwin O. Magomere, Shirley A. Onyango, Daibin Zhong, Linda E. Amoah, Andrew K. Githeko, Yaw A. Afrane, Guiyun Yan

**Affiliations:** 1 West Africa Centre for Cell Biology of Infectious Pathogen (WACCBIP), University of Ghana, Accra P.O. Box LG 54, Ghana; 2 Sub-Saharan Africa International Centre of Excellence for Malaria Research, Homabay P.O. Box 199-40300, Kenya; 3 Centre for Global Health Research, Kenya Medical Research Institute, Kisumu P.O. Box 20778-00202, Kenya; 4 Program in Public Health, College of Health Sciences, University of California, Irvine, CA 92697, USA; 5 Noguchi Memorial Institute for Medical Research, University of Ghana, Accra P.O. Box LG 581, Ghana; 6 Department of Medical Microbiology, University of Ghana Medical School, College of Health Sciences, University of Ghana, Accra P.O. Box 4236, Ghana

**Keywords:** *Anopheles funestus*, western Kenya, *COII*, genetic diversity, gene flow

## Abstract

**Simple Summary:**

*An. funestus* is a major vector of human malaria and is responsible for high transmissions in sub-Saharan Africa. In the malaria endemic region of western Kenya, it has adapted and colonized different ecological niches owing to its high resistance to pyrethroids and changing breeding environments. The genetic basis of its ecological adaptations to various settings, which could enrich our understanding of how the population is structured or segregated, is poorly understood. This study sought to evaluate the population structure and genetic diversity of *Anopheles funestus* in different landscapes in western Kenya. To achieve this, the cytochrome oxidase subunit II gene (*COII*) was PCR-amplified and sequenced. This study revealed an excess of low-frequency variations that are likely due to population expansion or possibly negative selection pressure. Our findings could serve as a guide for future genomic research to facilitate the design of control strategies.

**Abstract:**

The mitochondrial marker, *COII,* was employed to assess the genetic structure and diversity of *Anopheles funestus,* a very important malaria vector in Africa that adapt and colonize different ecological niches in western Kenya. Mosquitoes were collected using mechanical aspirators in four areas (Bungoma, Port Victoria, Kombewa, and Migori) in western Kenya. Following morphological identification, PCR was used to confirm the species. The *COII* gene was amplified, sequenced, and analyzed to determine genetic diversity and population structure. A total of 126 (Port Victoria-38, Migori-38, Bungoma-22, and Kombewa-28) sequences of *COII* were used for population genetic analysis. *Anopheles funestus* had a high haplotype diversity (Hd = 0.97 to 0.98) but low nucleotide diversity (Π = 0.004 to 0.005). The neutrality test revealed negative Tajima’s *D* and Fs values indicating an excess of low-frequency variation. This could be attributed to either population expansion or negative selection pressure across all the populations. No genetic or structural differentiation (Fst = −0.01) and a high level of gene flow (Gamma St, Nm = 17.99 to 35.22) were observed among the populations. Population expansion suggests the high adaptability of this species to various ecological requirements, hence sustaining its vectorial capacity and malaria transmission.

## 1. Introduction

Malaria is a public health problem in Sub-Saharan Africa and is spread mainly through members of the *Anopheles funestus* group and the *Anopheles gambiae* species complex [1]. The *An. funestus* group comprises five subgroups, of which three of these subgroups contain at least thirteen (13) species that are identified in various ecological niches across Africa [2]. *Anopheles funestus sensu stricto* (s.s.) (hereafter *An. funestus*) belongs to the *Funestus* subgroup, which has seven members, namely, *An. funestus, An. aruni, An. vaneedeni, An. funestus-like, An. confuses, An. longipalpis type C* and *An. parensis* [2,3]. Of this group, *An. funestus* is a major vector that is responsible for high malaria transmission in sub-Saharan Africa. Three of the members of the *An. funestus* group, *An. funestus, An. Rivulorum, and An. Leesoni* were found sympatrically in various ecological zones in Kenya [4], Sudan [5], and Nigeria [6,7], suggesting that they had effective reproductive isolation mechanisms.

While most of the species of the *Anopheles funestus* group can be found only in certain geographical areas in Africa, *An. funestus* has a wide range of geographical distributions across various climatic types. This vector remains one of the most devastatingly efficient human malaria vectors exhibiting consistently notorious anthropophilic (preferring human habitation), anthropophagic (biting humans), endophilic (indoor resting), and endophagic (indoor biting) behaviors [8,9]. Its capacity to transmit human malaria far outpaced *Anopheles gambiae* and *Anopheles arabiensis* in some endemic areas in Africa [8,10].

*An. funestus* can adapt and colonize different ecological niches owing to its high resistance to insecticides and changing breeding environments. A previous study in Kenya revealed that *An. funestus* breeds in various habitats and co-breeds with *An. gambiae sensu lato*, *Culex spp.,* and other vectors in the same habitats [11]. The vector survival rate, behavior, ecology, vectorial capacity, and host–pathogen interactions are all affected by external environmental stress, including temperature changes, land-use changes, host migration, and insecticide use [12]. These environmental factors have been demonstrated to influence mosquito population selection [12]. A study using microsatellite markers identified three genetically different *An. funestus* clusters namely: FUN1, FUN2, and FUN3 in Kenya [13]. The largest cluster (FUN1) was identified in samples collected from the Rift Valley and Western regions, while FUN2 and FUN3 were identified in coastal region samples.

The mitochondrial DNA (mtDNA) is sensitive to genetic drift and has a high copy of numbers, and highly conserved primer binding and ease of amplification making it a good marker for interpreting molecular taxonomy, phylogenetic relationships, population structure, and genetic diversity [14]. Indeed, mtDNA markers have been utilized to study the genetic variances and evolutionary relationships of many mosquito species, as well as to correctly quantify the gene flow and changes between populations [15,16].

As a mitochondrial marker, *COII* has a number of properties such as maternal inheritance, which is devoid of recombination, intraspecific polymorphism, a higher level of differentiation between populations, and small effective population sizes which make it a good marker for studying genetic diversity, gene flow, and population structure [17]. Mitochondrial genetic diversity and molecular phylogeny are becoming increasingly important in mosquito research [18].

The population structure and genetic diversity of *An. funestus* might influence its adaptation and efficiency of malaria transmission in western Kenya. Delineating the fine-scale population structure of vectors might be useful for investigating the genetic basis of speciation and local adaptation processes. Moreover, understanding the gene flow among *An. funestus* populations could help to assess their movement in natural populations and, therefore, how the populations are segregated. This study was designed to investigate the genetic structure, diversity, and gene flow of a major vector, *An. funestus* in a malaria-endemic region of western Kenya using the mitochondrial marker *COII*.

## 2. Materials and Methods

### 2.1. Mosquito Sampling

**Figure 1:**
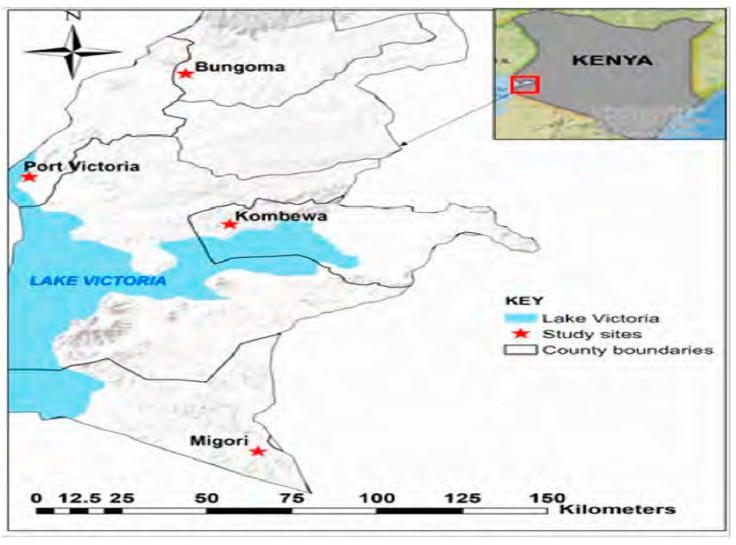
Map of study areas in western Kenya.

*Anopheles funestus* mosquitoes were collected from four malaria transmission areas (Port Victoria, Migori, Bungoma, and Kombewa) in four counties in western Kenya from November 2020 to October 2021. The sample collection sites are shown in Figure 1. Adult mosquitoes were sampled indoors using pyrethrum spray catches and mechanical aspirators. All mosquitoes were morphologically identified using morphological keys [19] and the *An. funestus sensu lato* was stored in 1.5 mL Eppendorf tubes containing cotton wool and silica gel [19]. Samples were stored at −20 °C for subsequent molecular analysis.

This map was prepared with ESRI ArcGIS Pro 2.8 using field survey results and publicly available datasets. The Open Database License, which is used to make the data available, was used to compile the material on the map from OpenStreetMap and the OpenStreetMap 115 Foundation.

### 2.2. DNA Extraction, PCR Amplification and Sequencing

Genomic DNA was extracted from the whole mosquito using the Chelex^®^-100 method [20]. *An. funestus*-specific PCR was conducted to confirm species using the species-specific primers (ITS2A/FUN) that could amplify the internal transcribed spacer region (ITS2) on the ribosomal DNA as described by [21]. The *COII* gene was amplified using forward (5′-TCTAATATGGCAGATTAGTGCA-3′) and reverse (5′-ACTTGCTTTCAG TCATCTAATG-3′) primers [17]. A final volume of 23 µL containing 3 µL genomic DNA, 0.5 µL each of forward and reverse primers, an 11.5 µL master mix with PerfeCTa^®^ ToughMix^®^ (5×), and 7.5 µL of PCR water were used. The PCR conditions included initial denaturation at 95 °C for 3 min followed by 35 cycles, denaturation at 95 °C for 15 s, and annealing at 41 °C for 30 s, with extension at 72 °C for 1 min and 30 s and final extension at 72 °C for 7 min. The resulting amplicons were separated by agarose gel electrophoresis in 1.5% *w*/*v* agarose gel stained with 2 µL smart glow. The SmartDoc imaging system (Accuris ^Tm^ instruments) was used to visualize the DNA bands and were imaged. All the amplicons were bidirectionally sequenced using the primers used in PCR amplification. Sequencing was performed on an ABI PRISM^®^ 3700 DNA Analyzer (Applied Biosystems, Foster City, CA, USA) platform using the 3730 BigDye^®^ Terminator v3.1 chemistry.

### 2.3. Data Analysis

The de novo assembly of paired raw reads was performed using the Geneious prime software version 2022.0.1 [22] and CLC Genomics Workbench [23]. Low-quality reads with a low base calling accuracy below 99% (Phred 20) were excluded from the analysis. The ClustalW algorithm in MEGA X was used for multiple sequence alignment [24]. The DnaSP Version 6.12.03 [25] was used to compute genetic diversity indices [haplotype diversity (Hd), the number of haplotypes (h), nucleotide diversity (Π), the number of segregating sites (S) and the mean number of pairwise difference (k)] and neutrality tests (Tajima’s D, Fu and Li’s D, Fu and Li’s F, and Fu’s Fs statistics). Gamma ST measurements, including an inbuilt algorithm with DnaSP software, were used to estimate the gene flow and genetic differentiation [26,27]. The analysis of molecular variance (AMOVA) was performed in Arlequin version 3.5.2 [28] to partition genetic variations among groups (Port Victoria, Migori, Bungoma, and Kombewa) and within groups. Population analysis with reticulate trees (PopART) version 1.7 [29] was used to infer haplotype networks.

The best-fitting nucleotide substitution model was estimated with the Akaike Information Criterion (AIC) and the Bayesian information criterion (BIC) implemented in MEGA version 11.0.13 [24]. The Bayesian phylogenetic analysis was performed by MrBayes v3.2.7 [30] using Markov chain Monte Carlo (MCMC) methods. The MCMC was run for 2,000,000 generations by sampling tree topologies every 1000 generations, after excluding the initial 25% as ‘burn-in’. To root the tree, *Anopheles gambiae, Aedes albopictus,* and *Culex pipiens pallens* were the “outgroup” (accession # MG930872, MG930866, KX383916, and KT851543). The previously reported *COII* sequences of *An. funestus* from western Kenya (GenBank accession: MT917174) and other locations in African countries, including southern Ghana (MT917179 and MT917180), northwestern Tanzania (MT917176 and MT917177), eastern Uganda (MT917175, MT917181, and MT917182), eastern Zambia (MT917178), and northern Malawi (MT917161), were also included in the phylogenetic tree. The 50% majority rule consensus tree was constructed with Bayesian posterior probabilities of the nodal supports. The output tree was visualized and edited with an online tool called the Interactive Tree of Life (iTOL) v5 [31].

## 3. Results

### 3.1. Genetic Diversity of An. funestus in Western Kenya

A total of 126 (Port Victoria-38, Migori-38, Bungoma-22, and Kombewa-28) amplicon sequences of COII were used for population genetic analysis. A total of 64 haplotypes were identified (Table S1), suggesting a high haplotype diversity in the populations (Hd = 0.97 to 0.98) albeit low nucleotide diversity (Π = 0.004) based on COII sequences (Table 1). Moreover, the statistical test of neutrality revealed significant negative Tajima’s D and Fs values indicating a deviation from a standard neutral model and population expansion with an excess of low-frequency variation likely due to population expansion (or possibly negative selection pressure). (Table 1). There was no significant difference in the observed nucleotide diversity across the four populations (X^2^ = 181.744, df = 189, *p* = 0.635).

### 3.2. Population Structure and Gene Flow

AMOVA results showed that there was no genetic differentiation across all four populations (Fst = −0.01). Specifically, our result revealed that there was no genetic differentiation between Port Victoria and Migori (Fst = −0.010), Bungoma and Kombewa (Fst= −0.016), Port Victoria and Bungoma (Fst = −0.020), Migori and Bungoma (Fst = −0.008), Port Victoria and Kombewa (Fst = −0.009), and Migori and Kombewa (Fst = −0.003) (Table 2). The lack of population structure was supported by a high level of gene flows across the four populations (Gamma St, Nm = 15.40), with the highest gene flow occurring between Port Victoria and Bungoma (Gamma St, Nm = 35.22). This was followed by Port Victoria and Migori (Gamma St, Nm = 30.25). The lowest gene flow was between Migori and Kombewa (Gamma St, Nm = 17.99) (Table 2).

### 3.3. Phylogenetic Relationships and Network Analyses

**Figure 2:**
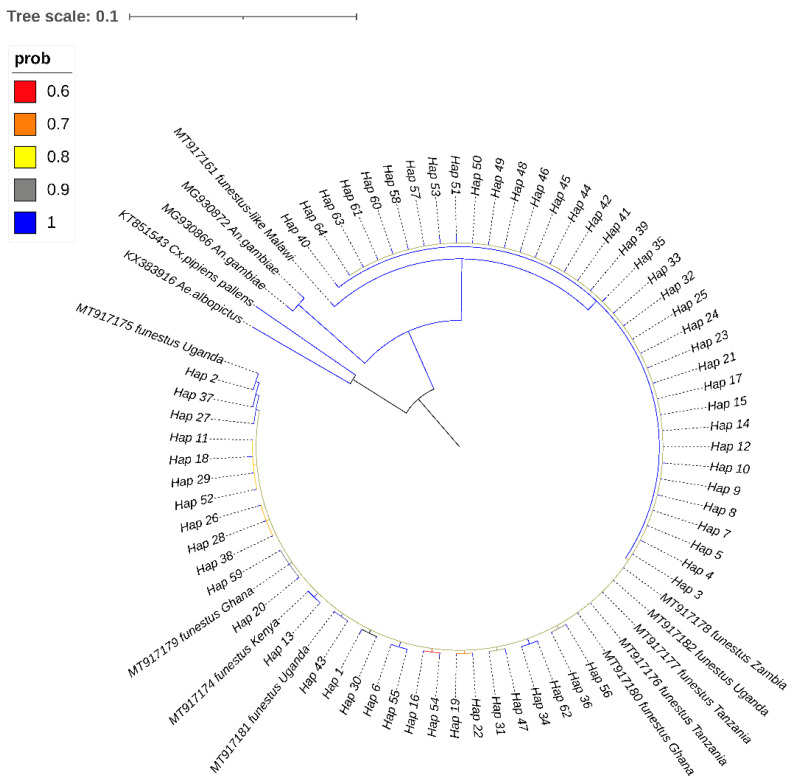
Bayesian phylogenetic tree inferred from cytochrome oxidase subunit II gene (*COII*). The 50% majority rule consensus tree was constructed using Markov chain Monte Carlo (MCMC) methods and posterior probabilities of the nodal supports are indicated by color lines where the green corresponds to a 100% support value).

The best-fit model for nucleotide substitution was identified by MEGA 11 as a Tamura 3-parameter with gamma-distributed rate heterogeneity (T92 + G) according to the Bayesian information criterion for *COII* haplotypes. The phylogenetic tree was inferred by Bayesian analyses with the standard deviation of split frequency values (*p* < 0.01). The phylogenetic estimates from the MCMC analyses strongly supported the monophyletic group (posterior probability = 1) (Figure 2). There was no clear clustering of haplotypes observed, and all the 64 identified haplotypes (Prob = 0.9983) in western Kenya shared a common ancestor with *An. funestus-like* (accession # MT917161) from Malawi, with the closest haplotype (Hap 40) coming from the Migori and Port Victoria populations, which border Tanzania and Uganda, respectively. *Anopheles funestus* samples from Port Victoria and Bungoma (Hap 2) shared a recent common ancestor with *An. funestus* (accession # MT917175) from Uganda.

**Figure 3:**
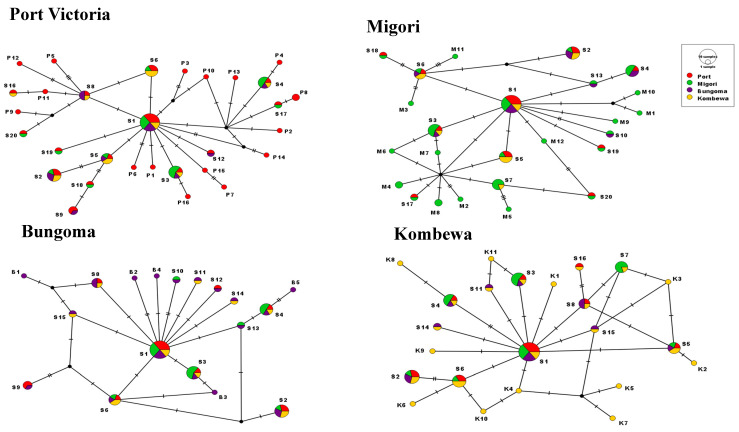
Haplotype distribution in the four study populations. S represents shared haplotypes across the four study sites, *p* represents haplotypes in Port Victoria only, M represents haplotypes in Migori, K represents haplotypes in Kombewa, and B represents haplotypes in Bungoma. Hatch marks represent the number of mutated sites resulting in particular haplotypes whereas the size of the circle corresponds to the haplotype frequency or numbers of the sample under that specific haplotype.

Haplotype networks were constructed using the median-joining method in PopART software. Out of the 64 distinct haplotypes identified in western Kenya, 30 (46.9%) were found in the Port Victoria population (Table 1). Twenty (31.3%) of the 64 haplotypes were found in each of the study populations. The median-joining haplotype network of the *COII* gene revealed the genealogy of each of the observed haplotypes, with haplotypes S1-S20 being shared across the study populations (Figure 3). In the four study areas, the most common haplotype was S1 (14/64, 21.9%), followed by S3 (7/64, 10.9%), S2 (7/64, 10.9%), S4 (6/64, 9.4%), S6 (6/64, 9.4%), and S8 (4/64, 6.3%). The distribution of the S1 haplotype among the 64 observed haplotypes was as follows: 8%, 6%, 5%, and 3% in Port Victoria, Migori, Bungoma, and Kombewa, respectively. The haplotype (S1) could be an ancestral haplotype (recent common ancestor) to *An. funestus* in western Kenya. Port Victoria and Bungoma populations had the most shared mutations (52.9%), followed by Migori and Port Victoria, as well as Kombewa, each at 50%. Port Victoria and Kombewa populations had the least shared number of mutated sites (42%). Nucleotide sequences of the 64 identified haplotypes were submitted to GenBank and assigned accession numbers ON931353-ON931416.

## 4. Discussion

This study revealed the population genetic structure of *An. funestus* population across four counties in western Kenya using a mitochondrial marker, *COII*. In the context of malaria control strategies, including the genetic alteration of vector species, information on the genetic diversity and population structure of vectors is critical [32]. In recent years, changes in the ecology of vector populations have been documented [11]. High tolerance to a variety of ecological niches, insecticide resistance, and vast geographic distribution make *An. Funestus* a highly adaptable dominant vector species [33]. Due to differences in the ecological zones in western Kenya, we anticipated that the barrier to gene flow could be due to variation brought about either by biotic or abiotic factors. Key biotic factors include Lake Victoria, climatic conditions, and landscape (highland versus lowland), resulting in gene flow and genetic diversity between the populations. Abiotic factors, including agriculture and the use of fertilizers and pesticides, could have selective pressure on the *An. funestus* population resulting in genetic diversity.

This study revealed the signature of population expansion, with weak population structure and high levels of gene flow among the population of *An. funestus* from areas with varied *p. falciparum* transmission intensities in western Kenya. Generally, *An. funestus* exhibited low nucleotide diversity, with Port Victoria exhibiting a higher number of haplotypes than other regions. In western Kenya, the high genetic diversity of the *An. funestus* population compared to coastal regions based on microsatellite markers was reported a decade ago [34]. The observed high number of haplotypes in Kombewa, Migori, and Port Victoria among this primary vector is consistent with high levels of *Plasmodial* transmission in these study areas compared to other malaria vectors [35,36]. Port Victoria, which is also proximal to Lake Victoria, had a significantly high number of haplotypes circulating in the area. Port Victoria is the main Lake Victoria transport corridor between Kenya, Lake Victoria Islands, and Uganda. It was earlier reported that small wooden boats played a significant role in the transportation of mosquitoes between the mainland and Lake Victoria Islands [37]. This might have played a role in facilitating high gene flow, resulting in a high number of haplotypes and haplotype diversity reported in that population. Port Victoria has also been documented to have a high malaria prevalence with vector control interventions ongoing [38]. The high gene flow observed in the populations could be due to migration and a lack of geographical barriers [15]. Mountain ranges, rivers, forests, and other physical barriers, in combination with climatic or biological obstacles, such as flight range and breeding grounds, may obstruct the gene flow between *Anopheles* populations. However, none of these factors served as a barrier to gene flow in the *An. funestus* population in western Kenya. The Rift Valley is known to serve as a barrier to gene flow [4]; nonetheless, our findings showed that there were no physical barriers to hamper gene flow among mosquito populations in western Kenya. Different breeding sites, mosquito migration, environmental changes, and human activities act to shape the genetic diversity of *An. funestus* populations [39].

Given the low Fst values and high Gamma St, Nm values, there was a strong indication of a high gene flow between populations as well as high breeding. Not only has a high gene flow reduced the heterozygosity between populations, but it has also resulted in most haplotypes being shared among the western Kenya region, contributing to a weak or lack of population structure. The shared haplotypes were observed between western Kenya and other African countries, suggesting that the genetic diversity of the *COII* gene might be not directly associated with geographical divisions. However, with the excess frequency of rare alleles, a genetic signature for population expansion could persist for a long period, masking any genuine ecological population or genetic structure that may exist [40]. The observed weak population structure, negative selection, and population expansion suggest that there was a free exchange of genes among *An. funestus* populations in western Kenya. The presence of a negative signature of selection on this gene is an indicator of purifying selection, which acts on the gene to preserve the genetic structure by eliminating deleterious mutations [41].

With evidence of purifying selection and population expansion (or possibly negative selection pressure), it is possible that evolutionary forces shaping the *An. funestus* population in western Kenya could be the usage of long-lasting insecticidal nets, indoor residual spraying, and insecticide use for agricultural activities, especially those affecting larval breeding sites [42,43]. The high number of haplotypes per study site and the population expansion observed in this study suggest the high adaptability of *An. funestus* to various ecological requirements, hence sustaining its vectorial capacity and malaria transmission.

## 5. Conclusions

This study has shown that the *An. funestus* population in western Kenya is under selection pressure leading to demographic expansion and the spread of variants through breeding among varied transmission sites in western Kenya. Population expansion (or possibly negative selection pressure) suggests there is high adaptability of this species to various ecological requirements hence allowing them to sustain their vectorial capacity and transmission of malaria.

## Figures and Tables

**Table 1:** Nucleotide diversity indices based on *COII* of *An. funestus* from four areas in western Kenya.

Populations	N	L	S	Π	h	Hd	Tajima’s		Fs		Fu and Li’s		Fu and Li’s	
							*D*	*p*	Statistics	*p*	*D*	*p*	*F*	*p*
Port Vic.	38	774	32	0.005	30	0.98	−1.86	<0.05	−31.51	0.000	−1.99	>0.10	−2.31	>0.05
Migori	38	774	26	0.004	25	0.97	−1.76	>0.05	−21.85	0.000	−2.75	<0.05	−2.86	<0.05
Bungoma	22	774	19	0.004	18	0.98	−1.64	>0.05	−16.42	0.000	−1.89	>0.10	−2.12	>0.05
Kombewa	28	774	17	0.004	23	0.98	−1.33	>0.10	−25.04	0.000	−1.06	>0.10	−1.34	>0.10
All populations	126		41	0.004	64	0.97	−1.81	<0.05	−32.91	0.000	−1.42	>0.10	−1.89	>0.05

N: sampled population, L: number of sites analyzed, S: segregating sites, Π: nucleotide diversity, h: number of Haplotypes, Hd: haplotype diversity, statistical significance (*p* < 0.05).

**Table 2:** *Anopheles funestus* population structure in the western Kenya region.

Populations	No. of Shared Mutations (%)	Dxy	Hs	Ks	Kxy	Gst	Gamma St		Fst		
							Value	Nm	Value	Nm	*p*-Value
Port Vic. vs. Migori	20/40 (50)	0.004	0.97	3.400	3.365	0.001	0.008	30.25	−0.010	−24.45	0.855
Bungoma vs. Kombewa	12/25 (48)	0.004	0.98	2.878	2.836	−0.006	0.013	19.43	−0.016	−16.04	0.892
Port Vic vs. Bungoma	18/34 (52.9)	0.004	0.98	3.389	3.222	−0.004	0.007	35.22	−0.020	−12.80	0.982
Migori vs. Bungoma	14/32 (43.8)	0.004	0.97	3.048	2.994	0.001	0.013	19.02	−0.008	−31.71	0.649
Port Vic vs. Kombewa	15/36 (41.7)	0.004	0.98	3.324	3.234	−0.003	0.011	23.43	−0.009	−28.29	0.838
Migori vs. Kombewa	15/30 (50)	0.004	0.97	3.015	2.985	0.0003	0.014	17.99	−0.003	−82.77	0.468

Dxy: the average number of nucleotide substitution per site between populations, Hs: weighted average of estimated haplotype diversities in the subpopulations, Ks: the number of synonymous substitutions per synonymous site, Kxy: the average number of nucleotide differences between populations, Gst: a measure of population differentiation, Nm: number of migrants.

## Data Availability

This study’s datasets are available in the online repository. The accession numbers for the haplotype sequence data submitted to GenBank are ON931353-ON931416.

## Supplementary Materials

The following is available online at https://www.mdpi.com/article/10.3390/insects14030273/s1, Table S1: Distribution of *COII* Haplotypes identified in the four *An. funestus* populations in western Kenya.
